# Ionizing terahertz waves with 260 MV/cm from scalable optical rectification

**DOI:** 10.1038/s41377-024-01462-w

**Published:** 2024-05-27

**Authors:** Hyeongmun Kim, Chul Kang, Dogeun Jang, Yulan Roh, Sang Hwa Lee, Joong Wook Lee, Jae Hee Sung, Seong Ku Lee, Ki-Yong Kim

**Affiliations:** 1 Advanced Photonics Research Institute, GIST, Gwangju, 61005 Korea; 2 Department of Physics and Optoelectronics Convergence Research Center, Chonnam National University, Gwangju, 61186 Korea; 3 Pohang Accelerator Laboratory, POSTECH, Pohang, 37673 Korea; 4 Center for Relativistic Laser Science, Institute for Basic Science, Gwangju, 61005 Korea; 5 Institute for Research in Electronics and Applied Physics; Department of Physics, University of Maryland, College Park, Maryland 20742 USA

**Keywords:** Terahertz optics, Nonlinear optics, Ultrafast photonics

## Abstract

Terahertz (THz) waves, known as non-ionizing radiation owing to their low photon energies, can actually ionize atoms and molecules when a sufficiently large number of THz photons are concentrated in time and space. Here, we demonstrate the generation of ionizing, multicycle, 15-THz waves emitted from large-area lithium niobate crystals via phase-matched optical rectification of 150-terawatt laser pulses. A complete characterization of the generated THz waves in energy, pulse duration, and focal spot size shows that the field strength can reach up to 260 megavolts per centimeter. In particular, a single-shot THz interferometer is employed to measure the THz pulse duration and spectrum with complementary numerical simulations. Such intense THz pulses are irradiated onto various solid targets to demonstrate THz-induced tunneling ionization and plasma formation. This study also discusses the potential of nonperturbative THz-driven ionization in gases, which will open up new opportunities, including nonlinear and relativistic THz physics in plasma.

## Abstract (web summary)

Intense terahertz pulses, produced from a large-area lithium niobate wafer via phase-matched optical rectification of 150-terawatt laser pulses, ionize atoms and molecules and turn them into plasma.

## Graphical abstract

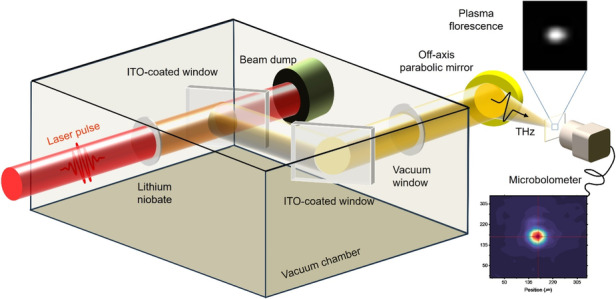

## Introduction

Recent advances in terahertz (THz) science and technology^1^ have opened up new opportunities to study novel phenomena in strong THz fields, such as THz-driven nonlinear spectroscopy^2–4^, phase transition^5,6^, impact ionization of semiconductors^7^, high harmonic generation^8,9^, and charged particle acceleration^10^. All these applications demand strong THz sources, and various methods have been developed to produce high-energy and/or high-intensity THz radiation. These include difference-frequency generation (DFG)^11–13^ or optical rectification (OR)^14–17^ of ultrafast laser pulses in nonlinear crystals, two-color laser mixing in gases^18–21^, laser-plasma interaction in liquids and solids^22–24^, coherent transition radiation (CTR) by ultrashort electron bunches^25^, laser-plasma accelerated electrons^26,27^, and more^28,29^.

**Fig. 1:**
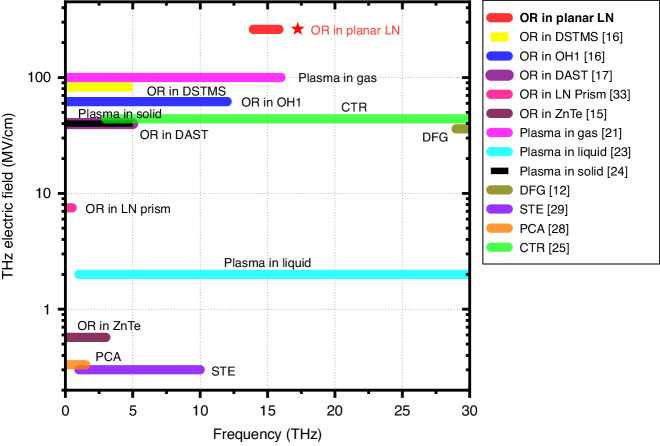
Strong-field THz sources. For each source, the THz electric field in MV cm^-1^ represents the highest reported value. The frequency range (color bar) covers the full width at 10% of the peak amplitude. High-power CO_2_ lasers operating near 30 THz are excluded, and because of this, 30 THz is considered the upper-frequency limit of the THz regime in this survey. Any sources producing less than 100 kV cm^-1^ are also omitted. The highest field strength demonstrated in this work is marked in red. OR optical rectification, LN lithium niobate, DFG difference-frequency generation, CTR coherent transition radiation, PCA photoconductive antenna, STE spintronic THz emitter

Some of the sources have produced THz electric field strengths in excess of 100 MV cm^−1^, as shown in Fig. 1. For instance, the DFG of two parametrically amplified laser pulses in GaSe has generated field strengths as high as 108 MV cm^−1^ at 53 THz and 36 MV cm^−1^ at 30 THz^12^. Two-color mixing of mid-infrared laser pulses in air has achieved 100 MV cm^−1^ ^21^. OR in organic crystals has yielded 83 MV cm^−1^ with DSTMS^16^, 62 MV cm^−1^ with OH1^16^, and 40 MV cm^−1^ with DAST^17^.

OR in lithium niobate (LN) has also been widely used due to its high nonlinearity^30^ and high damage threshold^31^, enabling the production of high-energy THz radiation. In addition, a tilted pulse front scheme can be adopted to achieve phase (or velocity) matching in a prism-shaped LN^14,30,32,33^. In this scheme, the generated THz wave propagates at the same velocity as the tilted laser pulse front inside the LN so that THz energy can continuously grow with the propagation distance. Recently, THz energy as high as 13.9 mJ has been produced by using two stacked cryogenically cooled LN prisms^33^. However, the radiation frequency produced from an LN prism is mostly peaked below 0.5 THz, which naturally leads to large focal spot sizes ( > mm), consequently limiting the peak THz field strength to at most 7.5 MV cm^−1^ until now^33^.

Recently, a new phase-matching condition has been discovered in LN, which does not require any pulse front tilting^34^. The working principle is that the velocity of THz waves is generally frequency-dependent and varies so large between two phonon resonance frequencies (7.4 THz and 18.8 THz in LN) that there exists a frequency at which the THz wave and the laser pulse driving OR propagate at the same velocity^34,35^. For an 800-nm optical pulse, this occurs at approximately 15 THz, where the refractive index of LN *n*_THz_ becomes equal to the optical group index of refraction *n*_g_ (*n*_THz_ = *n*_g_ = 2.3). The resulting phase-matched radiation at 15 THz, in principle, can be tightly focused due to its relatively short wavelength of 20 μm, potentially producing strong electromagnetic fields at the focus. This scheme uses a planar LN crystal and is favorable for scaling up the output THz energy^36^.

In this study, we demonstrate high-energy, high-intensity THz generation from large-area planar LN crystals by utilizing phase-matched OR at 15 THz. We also characterize the generated THz waves by their energy, beam profile, spectrum, and pulse duration. Such measurements allow us to determine the peak intensity or field strength of the THz waves at the focus. We also perform numerical simulations on THz wave generation in LN to accurately reproduce the THz pulse shape and spectrum, as well as to understand what thickness of LN can yield the highest THz energy. Finally, we experimentally demonstrate THz-induced ionization by focusing high-power 15-THz waves onto various solid materials, including metals, semiconductors, and polymers.

## Results

### Experiment

**Fig. 2:**
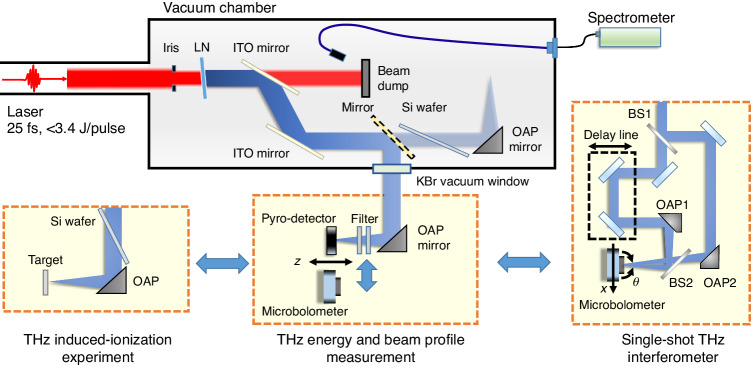
Experimental setup for generating, characterizing, and focusing 15 THz waves to induce THz-driven ionization. The energy and spatial beam profile of the THz waves (blue beams) produced from a 3-inch-diameter LN wafer are measured with a pyroelectric detector and a microbolometer (middle dashed box). The temporal profile and spectral power are characterized by a single-shot THz interferometer (right dashed box). The generated THz waves are focused onto a solid target to demonstrate THz-induced ionization (left dashed box)

A schematic of our setup is shown in Fig. 2. In this experiment, we used a 150-TW Ti:sapphire laser capable of producing <3.4 J, >25 fs pulses at a repetition rate of 5 Hz^37^. The laser was linearly polarized (horizontal) and had a center wavelength of 800 nm with a 78-nm full width at half-maximum (FWHM). The laser pulses after the pulse compressor were directed to the experimental vacuum chamber (see Fig. 2) through a series of vacuum tubes to avoid air-induced self-nonlinearities. The laser was operated in a single-shot mode to minimize cumulative degradation of the compressor gratings due to thermal loading.

Inside the vacuum chamber, the laser beam was apertured by a 60-mm-diameter iris diaphragm and then incident onto a 3-inch-diameter LN crystal wafer for THz generation via OR. The extraordinary axis of the LN wafer was aligned parallel to the laser polarization for maximal THz generation. The LN wafer was slightly tilted to prevent laser back reflection toward the laser system. After the LN wafer, the THz waves were decoupled from the IR laser by a pair of windows coated with indium tin oxide (ITO) layers (250-nm thickness) on the front reflection surfaces. This ITO decoupler was tilted at a Brewster angle of 56°, providing minimal reflectance of <0.0001% at 800 nm and maximal reflection of 64% at 15 THz^34^. For characterization, the generated THz waves were intercepted by a metallic 45° mirror and directed out of the vacuum chamber through a potassium bromide (KBr) window of 3-inch diameter and 25-mm thickness and then focused by a gold-coated, off-axis parabolic mirror (OAP) of 3-inch diameter and 4-inch focal length. More details on THz characterization are provided in the Materials and methods.

### THz energy

**Fig. 3:**
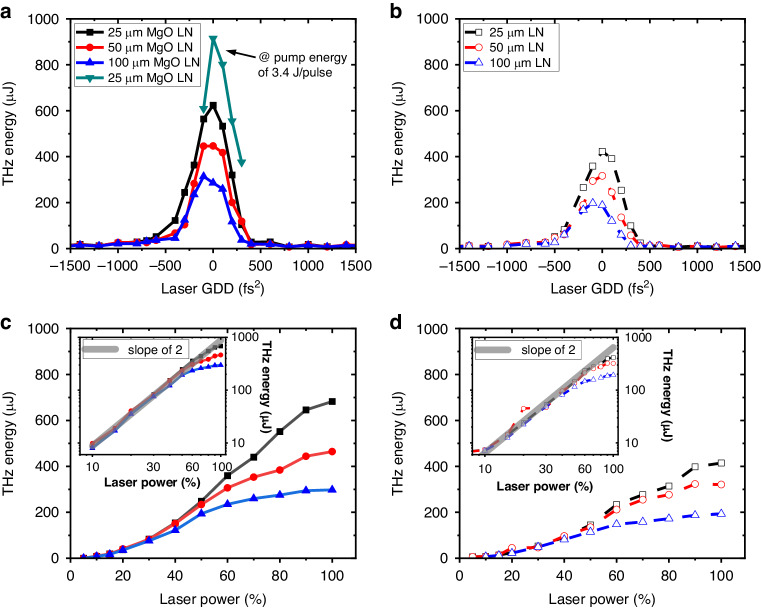
THz output energy scaling. **a**, **b** THz output energy measured as a function of input laser GDD at fixed laser energy of 2.3 J (except the green scatters taken at 3.4 J), obtained from *x*-cut LNs (**a**) with and (**b**) without 5% MgO doping. **c**, **d** THz output energy versus input laser energy obtained from *x*-cut LNs (**c**) with and (**d**) without 5% MgO doping at the optimal GDD for each LN. The insets show log-log plots. The gray lines represent a log-log slope of 2

For THz generation, large-area *x*-cut congruent LN wafers were used. The LN wafers had a diameter of 3 inches and thicknesses of 25 μm, 50 μm, and 100 μm, either doped or undoped with 5% MgO. The THz energies generated from the LN wafers were measured using a pyroelectric detector, as illustrated in Fig. 3.

Figure 3a, b plot the output THz energy generated from the MgO-doped and undoped LN wafers, respectively, as a function of the group delay dispersion (GDD) of the incident laser pulse. Here, the incident laser energy on the LN wafers was fixed at 2.3 J except the scan marked with green scatters (3.4 J). The voltages measured by the pyroelectric detector were converted to THz energies using the detector’s responsivity of 0.255 μJ V^−1^ at 15 THz. This responsivity was obtained by using a femtosecond 800-nm laser as a calibration source, together with relative frequency-dependent correction data provided by the detector vendor (see Figs. S1 and S2 in the Supplementary Material). The THz energy is retrieved after taking into account the transmission of the filters and attenuators used in the beam path, including the ITO decoupler (64% transmission), KBr window (83%), 7.3-μm longpass filter (49%), 15.7-THz bandpass (42%), 10-mm-thick Si window (14.4%) and Si wafers (50.6, 58.8, and 54.1%). Note that the THz energy plotted in Fig. 3 represents the energy estimated immediately after the LN emitter.

Figure 3a, b also show that the output THz energy peaks near zero GDD, indicating that the shorter laser pulse produces more THz energy at a fixed laser energy. Additionally, with increasing LN thickness, the optimal GDD shifts to a more negative value. This is due to a combination of the intrinsic positive dispersion (430 fs^2^ mm^−1^ at 800 nm) in bulk LN and nonzero third-order dispersion (TOD) present in the laser pulses. In addition, Fig. 3a, b show that a thinner LN wafer produces more THz energy. This is mostly due to THz absorption in LN (see the simulation section for details). In addition, MgO-doped LN wafers yield approximately 1.4 ~ 1.6 times more THz energy than undoped ones for the same thickness. This is because MgO doping can enhance not only the damage threshold^31^ but also the nonlinear coefficients d_33_ and d_31_^38,39^. The green scatter at zero GDD represents the highest THz energy, reaching up to 0.91 mJ, obtained with a 25-μm-thick MgO-doped LN wafer at a pump energy of 3.4 J.

Figure 3c, d show how the output THz scales with increasing laser energy for three thicknesses of MgO-doped and undoped LN wafers. In our experiment, the laser energy was varied by rotating a half-wave plate placed before the laser pulse compressor, with the optimal GDD value fixed for each LN. Since OR is a second-order nonlinear effect based on χ^(2)^, the output THz energy is expected to scale quadratically with the input laser energy. This quadratic dependence is observed at relatively low laser power, but it slowly deviates with increasing laser power, as shown in the log-log (inset) plots. The signal even saturates with the 100-μm-thick LN wafer. This deviation and saturation is due to three-laser-photon absorption and the cascading effect in LN^34,35,40,41^. The cascading effect broadens the pump laser spectrum and can weaken the OR process, as detailed in the simulation section.

### THz beam profile

**Fig. 4:**
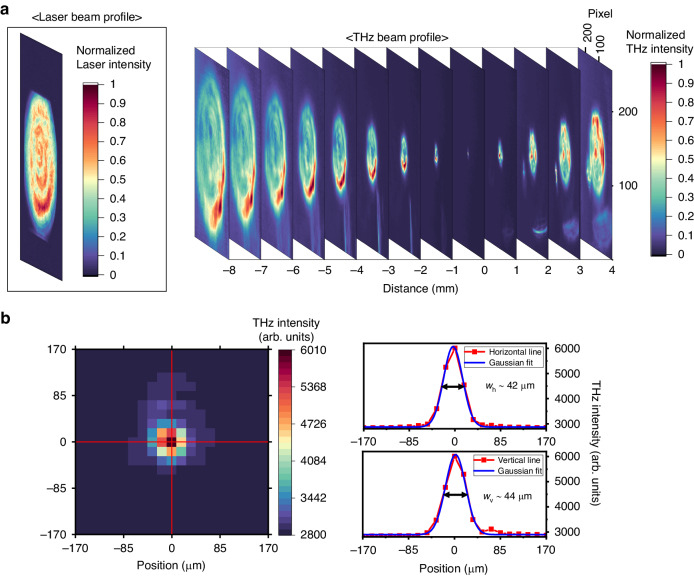
THz beam profiling. **a** THz beam profiles captured at various positions along the propagation when focused by an OAP mirror. **b** THz beam profile at the focus and its lineouts and Gaussian fits in the horizontal (right top) and vertical (right bottom) directions

For spatial characterization, the generated THz waves were focused by the OAP mirror onto a microbolometer camera (see Fig. 2). A 7.3-μm longpass filter and a 15-THz bandpass filter were inserted in the beam path to block any unwanted waves. One or more Si wafers were also used to attenuate the THz waves and prevent possible THz-induced damage to the camera. The LN wafer used here was a 25-μm-thick MgO-doped wafer. Figure 4a presents the evolution of the THz beam profiles captured along the propagation direction at an incremental distance of 1 mm from the focus (*z* = 0 mm). The beam diameter decreases almost linearly as the camera’s position approaches the focal point while maintaining its circular shape. Locally bright edges, however, appear in nearly all profiles, which are attributed to a nonuniform intensity distribution of our laser pulses (see Fig. 4a and Fig. S3).

Figure 4b shows the THz beam profile at the focus. The horizontal and vertical lineouts are well fitted with Gaussian profiles, with focal sizes of *w*_*h*_ = 42 ± 1 μm and *w*_*v*_ = 44 ± 1 μm in FWHM (see Fig. S4 for fitting results). The geometric average is *w*_FWHM_ = $\sqrt{{w}_{h}{w}_{{v}}}$ = 43 ± 1 μm in FWHM or *w* = 37 ± 1 μm (radius at 1/*e*^2^). This is ~1.7 times larger than the diffraction-limited value of *w* = 22 μm, estimated from *f*/# = 1.7 and the central wavelength of 20 μm.

### THz temporal and spectral profiles

To obtain the temporal profile of the generated THz radiation, we utilized a single-shot THz interferometer^42^. In our single-shot scheme, the interference between ${E}_{1}(t)$ and ${E}_{2}(t-\tau )$, representing two THz fields separated by *τ* in time, yields a time-integrated intensity given by^42^

1 $$I(\tau )={\int }_{\!-\infty }^{\infty }{|{E}_{1}(t)+{E}_{2}(t-\tau )|}^{2}dt={I}_{1}+{I}_{2}+\varDelta I(\tau )$$

where ${I}_{1}={\int }_{\!-\infty }^{\infty }{|{E}_{1}\left(t\right)|}^{2}{dt}$, ${I}_{2}={\int }_{\!-\infty }^{\infty }{|{E}_{2}\left(t\right)|}^{2}{dt}$, and $\Delta I\left(\tau \right)={\int }_{\!-\infty }^{\infty }{{E}_{1}\left(t\right)}^{* }{E}_{2}\left(t-\tau \right){dt}+{\int }_{\!-\infty }^{\infty }{E}_{1}\left(t\right){E}_{2}^{* }\left(t-\tau \right){dt}$. The term $\Delta I\left(\tau \right)$ represents a cross-correlation function that depends on $\tau$. The Fourier transform of the cross-correlation function can be expressed in the frequency domain as

2 $$\widetilde{I}\left(\omega \right)={\widetilde{E}}_{1}\left(\omega \right){\widetilde{E}}_{2}^{* }\left(\omega \right)+{\widetilde{E}}_{1}^{* }\left(\omega \right){\widetilde{E}}_{2}\left(\omega \right)$$

which provides the spectral information $\sqrt{{I}_{1}(\omega ){I}_{2}(\omega )}$ of the THz waves. For two identical THz waves, $E(t)$ and $E(t-\tau )$, separated by $\tau$ in time, the Fourier transform of the autocorrelation term provides the spectral power $I(\omega )$.

In our geometry, two identical THz beams are incident on the microbolometer camera with a crossing angle of $\theta$ (see Fig. 2). The relative time delay $\tau$ can be mapped onto the microbolometer’s sensor surface as

3 $$\tau =2x\sin (\theta /2)/c$$

where *x* is the distance parallel to the sensor surface and *c* is the speed of light. The crossing angle $\theta$ can be deduced from the mapping ratio $\tau /x$, which can be experimentally measured by scanning the delay stage. In our setup, the entire interference fringes were translated by 35 pixels when a 400-fs time delay was imposed. This gives $\tau /x$= $11.4$ fs pixel^−1^ and $\theta$=11.6°.

**Fig. 5:**
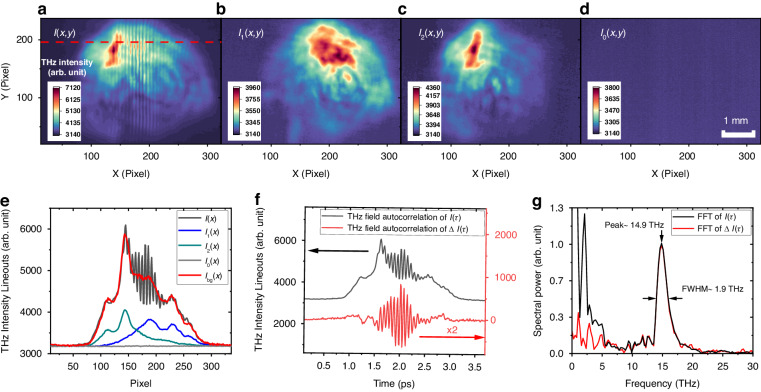
THz pulse characterization in time and frequency. Microbolometer images obtained with (**a**) two THz replica beams turned on, (**b**) the first beam on only, (**c**) the second beam on only, and (**d**) both beams off. **e** Horizontal lineouts from each image, where *I*_bg_(*x*) represents the background signal, ${I}_{{bg}}(x){=C}_{1}{I}_{1}\left(x\right)+{C}_{2}{I}_{2}\left(x\right)-{C}_{0}{I}_{0}\left(x\right)$. **f** THz autocorrelation signals, *I*(*τ*) (black line) and background-free Δ*I*(*τ*) (red line), and (**g**) their corresponding THz spectra obtained by Fourier transformations

Figure 5a–d present an example of THz beam profiles obtained in four distinct imaging modes. Figure 5a displays an intensity profile $I\left(x,y\right)$ produced when both THz beams were unblocked. It clearly shows an interference pattern with vertical fringes. Figure 5b, c show individual THz intensity profiles, ${I}_{1}\left(x,y\right)$ and ${I}_{2}\left(x,y\right)$. Both images show no interference patterns, as expected. Figure 5d represents a noise intensity ${I}_{0}\left(x,y\right)$ captured with all beams blocked, representing the background noise. The autocorrelation of the THz field can be obtained from $\Delta I\left(x,y\right)=I\left(x,y\right)-{I}_{{bg}}\left(x,y\right)$, where ${I}_{{bg}}\left(x,y\right)$ is the background signal corresponding to ${I}_{1}+{I}_{2}$ in Eq. (1). To take into account shot-to-shot variations in the camera’s counts, we model the background signal as ${I}_{{bg}}(x,y){=C}_{1}{I}_{1}\left(x,y\right)+{C}_{2}{I}_{2}\left(x,y\right)-{C}_{0}{I}_{0}\left(x,y\right)$, where ${C}_{1}$, ${C}_{2}$, and ${C}_{0}$ are the scaling coefficients that minimize the quasi-DC frequency in the Fourier transform of ${I}_{{bg}}(x,y)$.

Figure 5e displays a horizontal lineout $I\left(x\right)$ (black line) selected from Fig. 5a. It also plots other horizontal lineouts, ${I}_{1}\left(x\right)$ (blue line), ${I}_{2}\left(x\right)$ (green line), ${I}_{0}\left(x\right)$ (gray line), and ${I}_{{bg}}\left(x\right)$ (red line), obtained with ${C}_{1}=1.75$, ${C}_{2}=2.70$ and *C*_0_ = 3.45. Figure 5f shows the resulting autocorrelation profile, $\Delta I\left(\tau \right)=I\left(\tau \right)-{I}_{{bg}}\left(\tau \right)$ (red line), in the time domain. Clearly, the autocorrelation exhibits multicycle oscillations. The corresponding power spectrum is obtained by a Fourier transformation and plotted in Fig. 5g (red line). The spectrum peaks at approximately 14.9 THz with a 1.9-THz FWHM. This bandwidth gives a Gaussian transform-limited pulse duration of 0.23 ps in FWHM.

Alternatively, the spectrum can be directly obtained from $I\left(x\right)$ without involving ${I}_{{bg}}\left(x\right)$. The black line in Fig. 5g represents the Fourier transform of $\,I\left(\tau \right)$ (black line) in Fig. 5f. This yields almost the same spectrum, except for the large low-frequency components due to an uncompensated background signal. Such a low-frequency region (<3 THz) is well distinguished from the frequency band of interest and can be ignored.

### Simulation of THz generation

**Fig. 6:**
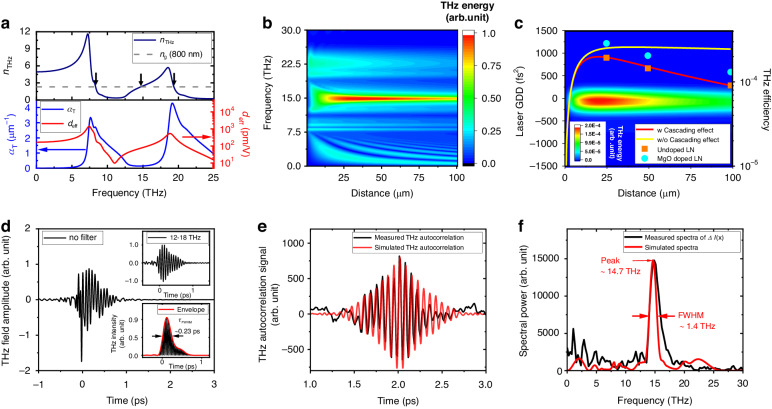
Simulation of THz pulse generation in LN. **a** Refractive index *n*_THz_ (top, black line), absorption coefficient *α*_*T*_ (bottom, blue line), and effective nonlinear coefficient *d*_eff_ (bottom, red line) of LN versus frequency. The dotted horizontal line (top) represents the group index of refraction *n*_g_ at 800 nm. The arrows indicate phase-matched frequencies. **b** Calculation of THz spectral power (false color) with increasing laser propagation distance in LN. **c** Simulated THz energy (false color) plotted as a function of the initial GDD and the propagation distance in LN. The co-plot is the simulated laser-to-THz conversion efficiency with (red line) and without (yellow line) the cascading and SPM effects. The scatters are our experimental results with (cyan circles) and without (orange squares) MgO doping in LN. **d** Simulated THz waveform after 25-μm propagation in LN. The insets show the waveform (top) after bandpass filtering at 12–18 THz and its corresponding intensity envelope (bottom, red line). **e** Comparison between simulated (red line) and measured (black line) THz autocorrelation signals and (**f**) their Fourier spectra

To simulate THz generation via OR, we solved one-dimensional (1-D) coupled forward Maxwell equations (FMEs), as detailed in the Materials and methods. First, the refractive index *n*_THz_, the linear absorption coefficient *α*_*T*_, and the effective nonlinear coefficient *d*_eff_ of LN are calculated as a function of THz frequencies^35^ and plotted in Fig. 6a. The dotted horizontal line indicates the group index of refraction *n*_g_ = 2.3 of the laser at 800 nm. Phase matching (*n*_THz_ = *n*_g_ = 2.3) occurs at three frequencies, 8.3 THz, 14.8 THz, and 19.3 THz, but only 14.8 THz is expected to be dominant because of its lowest absorption coefficient. *d*_eff_ is also relatively large at 14.8 THz (*d*_*eff*_ ≈ 81 pm V^−1^) to produce high-energy THz radiation.

Figure 6b shows the calculation of the output THz spectral power with an increasing propagation distance within LN. The simulation was performed by setting the input laser pulse to have an energy of 2.3 J, a fluence of 0.08 J cm^−2^, a Gaussian spectrum with a FWHM of 78 nm centered at 800 nm, and initial GDD and TOD values of 0 fs^2^ and 1000 fs^3^, respectively. It shows that a noticeable peak appears in the output THz energy at approximately 15 THz when the propagation distance reaches approximately 25 μm. As the distance increases further, the output energy at 15 THz then decreases.

This trend is more evident in Fig. 6c, which plots the cumulative THz energy at 10-20 THz as a function of the initial laser GDD and the propagation distance in LN. The result shows that 15-THz radiation can be most efficiently produced from a thin (<25 μm) LN with the input laser pulse duration as short as possible, i.e., GDD ≈ 0 fs^2^. Figure 6c also plots the expected THz conversion efficiencies (solid lines). As the propagation distance increases in LN, the output THz energy initially rises, reaching its peak at 18 ~ 25 μm, and then either decreases or saturates depending on whether the cascading and self-phase modulation (SPM) effects (see the simulation section in Materials and methods) are included (red line) or not (yellow line). Together with dispersion, the cascading effect can broaden the laser pulse duration, thereby weakening the OR process. The SPM effect, however, is negligible in our simulation due to its small nonlinear refractive index in LN^34^. Even without these effects, the THz yield saturates because of linear absorption (~0.14 μm^−1^ at 15 THz), which fundamentally limits the conversion efficiency. The scatters in Fig. 6c are our experimental data obtained with three thicknesses of LN (25, 50, and 100 μm), with (cyan circles) and without (amber squares) MgO doping. The data agree reasonably well with the simulation results.

Figure 6d shows the simulated THz waveform after a 25-μm propagation in LN. It exhibits multicycle 15-THz oscillations on top of low-frequency oscillations at <5 THz^35^. The waveform, extracted after 12 ~ 18 THz bandpass filtering, is shown in the top inset. The corresponding intensity envelope (red line) is shown in the bottom inset and provides a pulse duration of ~0.23 ps in FWHM. Figure 6e presents the autocorrelation trace (red line) of the simulated THz waveform, together with the measured waveform (black line) for comparison. Their corresponding power spectra are obtained by Fourier transformations and shown in Fig. 6f. The simulated spectrum peaks at 14.7 THz with a bandwidth of 1.4 THz in FWHM, reasonably consistent with the measured peak at 14.9 THz with an FWHM of 1.9 THz. The bandwidths show a 36% discrepancy, which is possibly due to the uncertainty of many parameter values used in the simulation.

### THz peak intensity and field strength

With an assumption of a Gaussian spatial and temporal pulse shape, the peak THz intensity is given by^43^,

4 $${I}_{T}=\frac{4\sqrt{\mathrm{ln}2}}{\pi \sqrt{\pi }}\frac{{\mathcal{E}}}{{w}^{2}{\tau }_{T}}$$

where ${\mathcal{E}}$ is the THz pulse energy, *w* is the THz beam waist at 1/*e*^2^, and *τ*_*T*_ is the THz pulse duration in FWHM. The highest THz energy achieved immediately after the LN wafer is 0.91 mJ. The energy drops to 0.47 mJ after the ITO decoupler (64% transmission) and the Brewster-angled Si wafer (81%) when focused inside the vacuum chamber. With ${\mathcal{E}}$ = 0.47 ± 0.05 mJ, *w* = 37 ± 1 μm, and *τ* = 0.23 ± 0.02 ps at the focus, the peak intensity of the focused THz waves is estimated to be *I*_*T*_ = (8.9 ± 1.3) × 10^13 ^W cm^−2^. The corresponding peak electric and magnetic field strengths are *E*_*T*_ = 260 ± 20 MV cm^−1^ and *B*_*T*_ = 87 ± 7 T, respectively (see Fig. S4 for detailed calculations of the uncertainties). To the best of our knowledge, this is the highest peak intensity or field strength demonstrated so far at 0.1 ~ 20 THz, as shown in Fig. 1.

An intense (>10^13 ^W cm^−2^) THz pulse, when focused into a neutral gaseous or solid medium, can ionize the constituent atoms or molecules and convert the medium into a plasma. In the plasma, the liberated electron acquires an average kinetic energy in the oscillating THz fields, i.e., the ponderomotive energy ${U}_{p}=\tfrac{1}{2}{m}_{e}\left\langle {v}_{o}^{2}\right\rangle ={e}^{2}{E}^{2}/\left(4{m}_{e}{\omega }^{2}\right)$, where *v*_*o*_ is the quiver velocity of the electron, and *m*_*e*_ and *e* are the electron mass and charge, respectively. In practical units, *U*_*p*_ [eV] = 9.33 × 10^−14^ *I*_*T*_[Wcm^−2^]*λ*^2^[μm]. At *I*_*T*_ = 8.9 × 10^13 ^W cm^−2^, the cycle-averaged kinetic energy of the electron can be as large as *U*_*p*_ = 3.2 keV. This energy is more than 5 orders of magnitude higher than the THz photon energy of 62 meV at 20 μm owing to the *Iλ*^2^-dependent scaling law. Such energy can be converted to produce multi-keV photons by high harmonic generation when the liberated electron recombines with its parent ion^44^. In addition, the normalized THz vector potential, ${a}_{0}=e{E}_{T}/\left({m}_{e}c\Omega \right)=v/c$, gets close to 1 when the classical velocity of the electron, $v$, approaches *c*, where relativistic effects can be dominant. In practical units, ${a}_{0}=0.86\lambda [{\rm{\mu }}{\rm{m}}]\sqrt{{I}_{T}[{10}^{18}{\rm{W\; c}}{{\rm{m}}}^{-2}]}$. In our case, *a*_0_ = 0.16, which gives a relativistic correction of 0.6% to the electron mass in the THz field. Similar to the ponderomotive energy, the intensity required to reach the relativistic regime can be substantially reduced thanks to the *Iλ*^2^ dependence.

Another important parameter relevant to ionization is the Keldysh parameter ${\gamma }_{K}=\sqrt{\frac{{U}_{i}}{2{U}_{p}}}=\sqrt{\frac{{t}_{{\rm{ion}}}}{T}}$, where *U*_*i*_ is the ionization potential energy of the atom or ion^45^. *γ*_*K*_ can also be expressed as the square root of the ratio of the ionization time *t*_ion_ to the THz period *T*. For a hydrogen atom (*U*_*i*_ = 13.6 eV) at *I*_*T*_ = 8.9 × 10^13 ^W cm^−2^, *γ*_*K*_ = 0.046. Such a small *γ*_*K*_ value implies that ionization occurs so rapidly, compared to the THz period of 67 fs, that the THz field suppressing the Coulomb potential barrier is seen as a static field by the bound electron. Under this circumstance, the bound electron can tunnel through the suppressed barrier and be freed. In this regime of *γ*_*K*_ < 1, tunneling ionization is a dominant mechanism over multiphoton ionization.

### THz-induced ionization and damage

For the study of intense THz-matter interactions, we first generated THz waves by using a 25-μm-thick LN crystal doped with 5% MgO. For simplicity, the target sample was placed outside the vacuum chamber, as shown in Fig. 2. The energy of the THz waves emitted from the LN was estimated to be 0.62 mJ (with 2.3 J pumping) but drops to 0.27 mJ on the target after the waves pass through the ITO decoupler, the KBr window, and the Brewster-angled Si wafer. In addition, the pulse is chirped by the KBr window, providing GDD = − 1.15 × 10^5^ fs^2^ at 15 THz. This stretches the THz pulse duration from 0.23 ps to 1.3 ps. With ${\mathcal{E}}$ = 0.27 mJ, *w* = 37 μm, and *τ* = 1.3 ps, the peak intensity of the focused THz waves is estimated to be *I*_*T*_ = 0.91 × 10^13 ^W cm^−2^ at the focus, and its corresponding THz field strength is *E*_*T*_ = 83 MV cm^−1^. The field strength is still high enough to induce tunneling ionization with the Keldysh parameter *γ*_*K*_ = 0.2 less than 1.

**Fig. 7:**
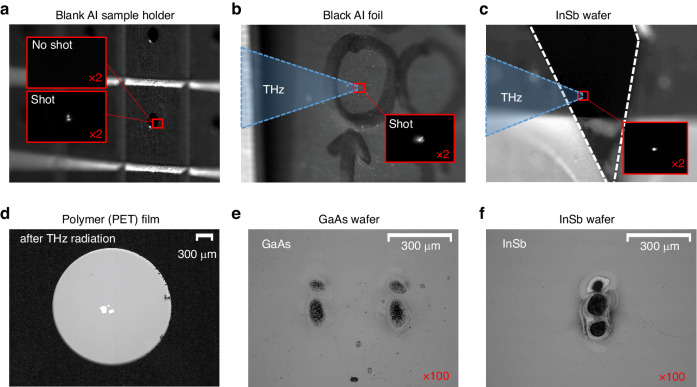
THz-induced ionization on solids. **a**–**c** Photographs of (**a**) aluminum holder, (**b**) black aluminum foil, and (**c**) InSb wafer targets irradiated by intense THz radiation, with the resulting plasma fluorescence captured by a CCD camera in each inset. **d**-**f** Microscope images of THz-induced damage made on (**d**) polymer (PET) film, (**e**) GaAs wafer, and (**f**) InSb wafer targets

As the first demonstration, a blank sample holder made of aluminum (Al) was exposed to a single THz pulse focused by the OAP mirror. Figure 7a shows a photograph of the aluminum target and a flash of plasma fluorescence captured by a charge-coupled device (CCD) camera (see Fig. S5 in Supplementary Material for raw photographs). Figure 7b shows a black aluminum foil exposed to an intense THz pulse and its resulting plasma emission. Figure 7c shows an InSb wafer together with its plasma emission. In these samples, the focused THz field is high enough to ionize the target by tunneling ionization and produce plasma on the target surface. For Al (*U*_*i*_ = 6.0 eV) and In (*U*_*i*_ = 5.8 eV), *γ*_*K*_ < 0.1, confirming that tunneling ionization is dominant over multiphoton ionization. The electrons initially created by tunneling ionization are then heated via inverse bremsstrahlung absorption of the trailing THz waves over a picosecond time scale. Simultaneously, it undergoes avalanche ionization under strong THz fields, ultimately emitting plasma fluorescence by recombination, electronic transition, and bremsstrahlung processes beyond the picosecond time scale. Additionally, plasma expansion, ablation, and structural deformation occur on nanosecond time scales.

**Fig. 8:**
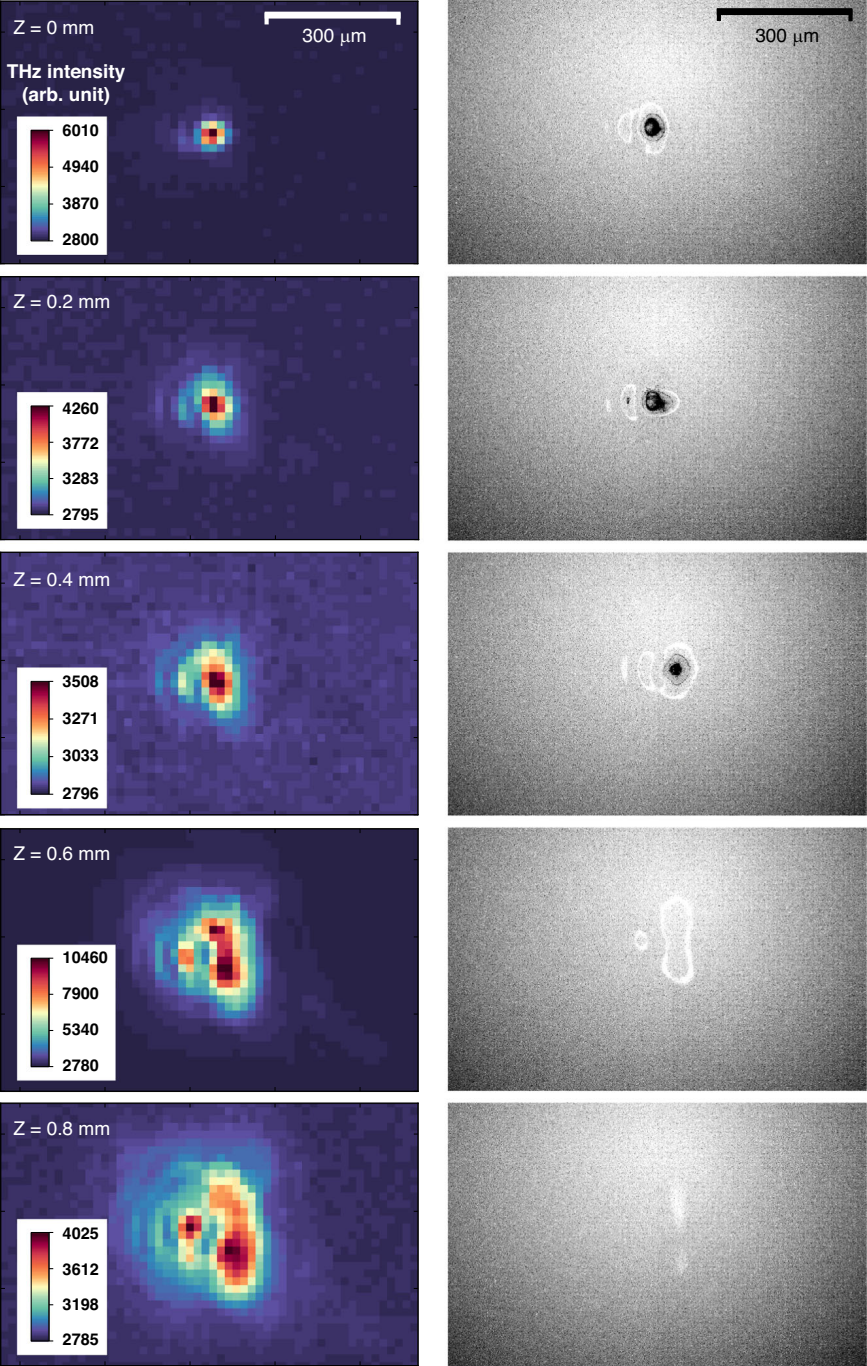
THz intensity-dependent ionization of GaAs. A series of THz beam profiles (left), captured by a microbolometer, and microscope images of the GaAs surface (right), both taken at the same position *z* along the THz beam propagation direction. The focal position is located at *z* = 0

As the fourth target, a free-standing polymer (PET) film was exposed to intense THz radiation at the focus. This resulted in irreversible damage, such as the removal or destruction of the film, as shown in Fig. 7d. It shows clear holes drilled through the PET film after the exposure. Figure 7e, f show THz-induced damage created on the surfaces of GaAs and InSb wafers after 5 consecutive shots of THz radiation. To further investigate the dependence of THz-induced damage on the intensity of irradiated THz waves, the GaAs sample was exposed to 5 shots of THz pulses with the sample position varied along the THz beam direction (*z*) with an increment of Δ*z* = 0.2 mm. Figure 8 shows a series of THz beam profiles (left) captured by the microbolometer and their corresponding images of THz-induced ablation (right) taken under a microscope. The damage patterns (right) closely resemble the intensity distributions of the irradiated THz radiation (left). The damage pattern, however, becomes less noticeable when the sample is positioned far away from the focal point, which drops the THz intensity and thus yields significantly less ionization.

## Discussion

In conclusion, we demonstrated the generation and characterization of mJ-level, intense THz fields, approaching 260 MV cm^−1^, at 15 THz from large-area phase-matched OR in LN by using a 150-TW Ti:sapphire laser. By testing various LN wafers with thicknesses of 25 μm, 50 μm, and 100 μm, also doped or undoped with 5% MgO, we found that the thinnest LN yields the largest THz energy owing to the linear absorption of THz and the cascade effect in LN. In addition, MgO-doped LNs produced 1.4 to 1.6 times more THz energy because of their higher nonlinearities than the undoped LNs. The spatial profiles of the produced THz waves, when focused by an OAP mirror, were characterized by a microbolometer camera, which measured the focal spot size as small as 43 μm in FWHM. The camera was also used in a single-shot THz interferometer, which allowed us to measure the field autocorrelation in time and its corresponding spectrum via a Fourier transformation. A numerical simulation was also conducted by solving 1-D coupled FMEs, and its results support the measured THz pulse spectrum centered at 14.9 THz with an FWHM of 1.9 THz. Furthermore, we demonstrated THz-driven tunneling ionization and subsequent collisional heating and avalanche ionization by focusing intense 15-THz waves onto aluminum, polymer and semiconductor samples.

Although the field strength of 260 MV cm^−1^ is the highest demonstrated thus far at 0.1 ~ 20 THz, there is still room for further enhancement. First, cryogenic cooling of the LN wafer can reduce the linear absorption of radiation at 15 THz, enabling the use of a thicker LN emitter to produce even higher THz energy. Second, our THz decoupler, based on ITO-coated windows and a Si Brewster-angled wafer, exhibits a throughput of 52%. A more efficient THz decoupler can deliver even higher THz energy onto the target. Third, tighter focusing can enhance the field strength even more. In our experiment, an OAP mirror with *f* = 4” was used to have space for various filters to be inserted for THz characterization. An OAP mirror with *f* = 3”, in principle, can be used to reduce the focal spot size and thus increase the field strength by up to 1.3 times. Finally, a larger planar LN can be used with more laser pumping to produce even higher THz energy. All these methods can assist in producing superstrong (~GV cm^−1^) THz field strengths at the focus. Such strong THz fields can allow us to not only study nonlinear effects in THz-produced plasmas but also utilize THz-driven ponderomotive forces for various applications, including THz-assisted harmonic and attosecond pulse generation in the optical regime^46^, multi-keV THz harmonic generation and even studying relativistic effects by THz-accelerated electrons.

## Materials and methods

### THz characterization

The energy of THz waves was measured by using a pyroelectric detector (Gentec, THz5D-MT-BNC). To block any residual laser beam leaking through the ITO decoupler, a 7.3-μm longpass filter was used in front of the pyroelectric detector. In addition, a 15.7-THz bandpass filter with a 2.4-THz FWHM was used to transmit THz waves within the band and cut off 0.1 ~ 3 THz waves also emitted from LN^36^. Such low-frequency THz waves were additionally cut off by the thick KBr window that strongly attenuates radiation at 40 − 300 μm^47^. One or more Si filters were placed in the beam path to attenuate THz energy and prevent any possible damage to the detector.

To characterize the spatial profiles of the generated THz waves, we employed a microbolometer imaging sensor (camera). The camera was a commercial uncooled vanadium oxide (VOx) microbolometer (FLIR, Tau2 336), featuring a resolution of 336 × 256 pixels with the pixel pitch of 17 μm. It was connected to a Camera Link expansion board (FLIR) and a frame grabber (NI, PCIE-1433) to capture 14-bit images at 60 frames per second. This microbolometer camera was previously used to characterize THz waves over a wide range of THz frequencies^19,42,48,49^.

To acquire the temporal and spectral profiles of the THz waves, we implemented a single-shot THz spectrometer^42^. This consisted of a modified Mach-Zehnder interferometer and a microbolometer camera, as illustrated in Fig. 2. Two identical high-resistivity silicon wafers served as beam splitters (BS1 and BS2) to divide and combine the incident THz beams with equal dispersion and intensities. One of the beams passed through a fixed optical path, while the other passed through an adjustable optical path using a delay stage. Two identical OAP mirrors of 3-inch diameter and 9-inch focal length were used to separately focus the split THz beams onto the microbolometer sensor. To create an interference fringe pattern on the camera, the two beams were crossed at an angle on the sensor plane. The autocorrelation obtained from the interference pattern allowed us to characterize the THz pulse duration and obtain the spectral power of the waves via a Fourier transformation.

For further investigation of THz-induced ionization and damage, the generated THz waves were focused onto solid materials such as aluminum, polymer films, and semiconductor wafers by an OAP mirror with a focal length of 4 inches. To block any residual IR laser in the beam and simultaneously allow maximal THz transmission, a high-resistivity (>10 kΩ), thin (0.675-mm thickness) Si filter was placed in the beam path at a Brewster angle of 67°, allowing maximal THz transmission of 81% at 15 THz^34^. A similar Brewster-angled Si filter was used inside the vacuum chamber to completely block the laser after the ITO decoupler.

### Simulation

We solved one-dimensional (1-D) coupled forward Maxwell equations (FMEs) for both THz and laser fields in the frequency domain^34,36^. This calculation fully resolves all carrier frequencies for both laser and terahertz pulses and is more powerful than nonlinear envelope equations commonly used to compute THz waves. Our 1-D coupled FMEs are given as

5 $$\begin{array}{l}\frac{\partial {E}_{T}(\varOmega ,\zeta )}{\partial \zeta }=-\frac{{\alpha }_{T}}{2}{E}_{T}(\varOmega ,\zeta )-jD(\varOmega ){E}_{T}(\varOmega ,\zeta )\\\qquad\qquad\;\;\;-j\frac{{k}_{T}{d}_{eff}}{{n}_{T}^{2}}{\int }_{\!0}^{\infty }{E}_{L}(\omega +\varOmega ,\zeta ){E}_{L}^{\ast }(\omega ,\zeta )d\omega\end{array}$$

6 $$\begin{array}{l}\frac{\partial {E}_{L}(\omega ,\,\zeta )}{\partial \zeta }=-\frac{{\alpha }_{L}}{2}{E}_{L}(\omega ,\zeta )-jD(\omega ){E}_{L}(\omega ,\zeta )\\\qquad\qquad\;\;\; -j\frac{{k}_{L}{d}_{eff}}{{n}_{L}^{2}}{\int }_{\!-\infty }^{\infty }{E}_{L}(\omega -\varOmega ,\zeta ){E}_{T}(\varOmega ,\zeta )d\varOmega\\\qquad\qquad\;\;\;-j\frac{{k}_{L}c{\varepsilon }_{0}{n}_{2}}{2}FT\{{|{E}_{L}(t,\zeta )|}^{2}{E}_{L}(t,\zeta )\}\end{array}$$

where *E* represents the electric field, and the subscripts *T* and *L* denote THz and laser, respectively. Ω and ω are the angular frequencies of the THz and laser fields, respectively, and $\zeta =z-{v}_{g}t$ corresponds to the coordinate moving at the group velocity of the laser. The wavevector *k* is given by $k\left(\omega \right)=n\left(\omega \right)\omega /c$. On the right side of Eqs. (5) and (6), the first terms ${\alpha }_{T}$ and ${\alpha }_{L}$ and the second terms $D\left(\Omega \right)=\Omega \left[n\left(\Omega \right)-{n}_{g}({\Omega }_{0})\right]/c$ and $D\left({\rm{\omega }}\right)={\rm{\omega }}\left[n\left({\rm{\omega }}\right)-{n}_{g}({{\rm{\omega }}}_{0})\right]/c$ account for the absorption and dispersion of the THz and laser fields, respectively. The last term in Eq. (5) represents the OR process, a radiation source. The effective nonlinear coefficient *d*_eff_ = *d*_eff_(Ω) is obtained from Ref. ^35^. In Eqs. (5) and (6), THz absorption by free carriers, induced by three-laser-photon absorption in LN, is not considered because of its small effect (13 cm^−1^) compared to single-photon absorption at 15 THz in LN (~0.14 μm^−1^)^34,41^. The third and last terms in Eq. (6) represent the cascading effect and the self-phase modulation (SPM) of the laser, respectively. The cascading term represents a THz-induced modulation of the laser pulse. The SPM term involves the use of a nonlinear refractive index of *n*_2_ = 10^−6^ cm^2^ GW^−1^ ^50^, with FT denoting a Fourier transformation. The input laser pulse was set to be Gaussian with a center wavelength of 800 nm and an FWHM of 78 nm.

### Supplementary information

Supplementary material

## Data Availability

The data that support the findings of this study are available from the corresponding authors upon reasonable request.
