# Supplementary material for: Ionizing terahertz waves with 260 MV/cm from scalable optical rectification
Source: Light Sci Appl. 2024 May 27;13:118. doi: 10.1038/s41377-024-01462-w (PMC11130333; doi:10.1038/s41377-024-01462-w)
Supplement: Supplementary file 1 — Supplementary material [file 41377_2024_1462_MOESM1_ESM.docx]

**Supplementary Material for**

**Ionizing terahertz waves with 260 MV/cm from scalable optical rectification**

Hyeongmun Kim^1,2^, Chul Kang^1,*^, Dogeun Jang^3^, Yulan Roh^1^, Sang Hwa Lee^4^, Joong Wook Lee^2^, Jae Hee Sung ^1,4^, Seong Ku Lee^1,4^, and Ki-Yong Kim^5,^**^**^**

^1^Advanced Photonics Research Institute, GIST, Gwangju 61005, Korea

^2^Department of Physics and Optoelectronics Convergence Research Center, Chonnam National University, Gwangju 61186, Korea

^3^Pohang Accelerator Laboratory, POSTECH, Pohang 37673, Korea

^4^Center for Relativistic Laser Science, Institute for Basic Science, Gwangju 61005, Korea

^5^Institute for Research in Electronics and Applied Physics; Department of Physics, University of Maryland, College Park, Maryland 20742, USA

^*^[iron74@gist.ac.kr](mailto:iron74@gist.ac.kr)

**^**^**[kykim@umd.edu](mailto:kykim@umd.edu)


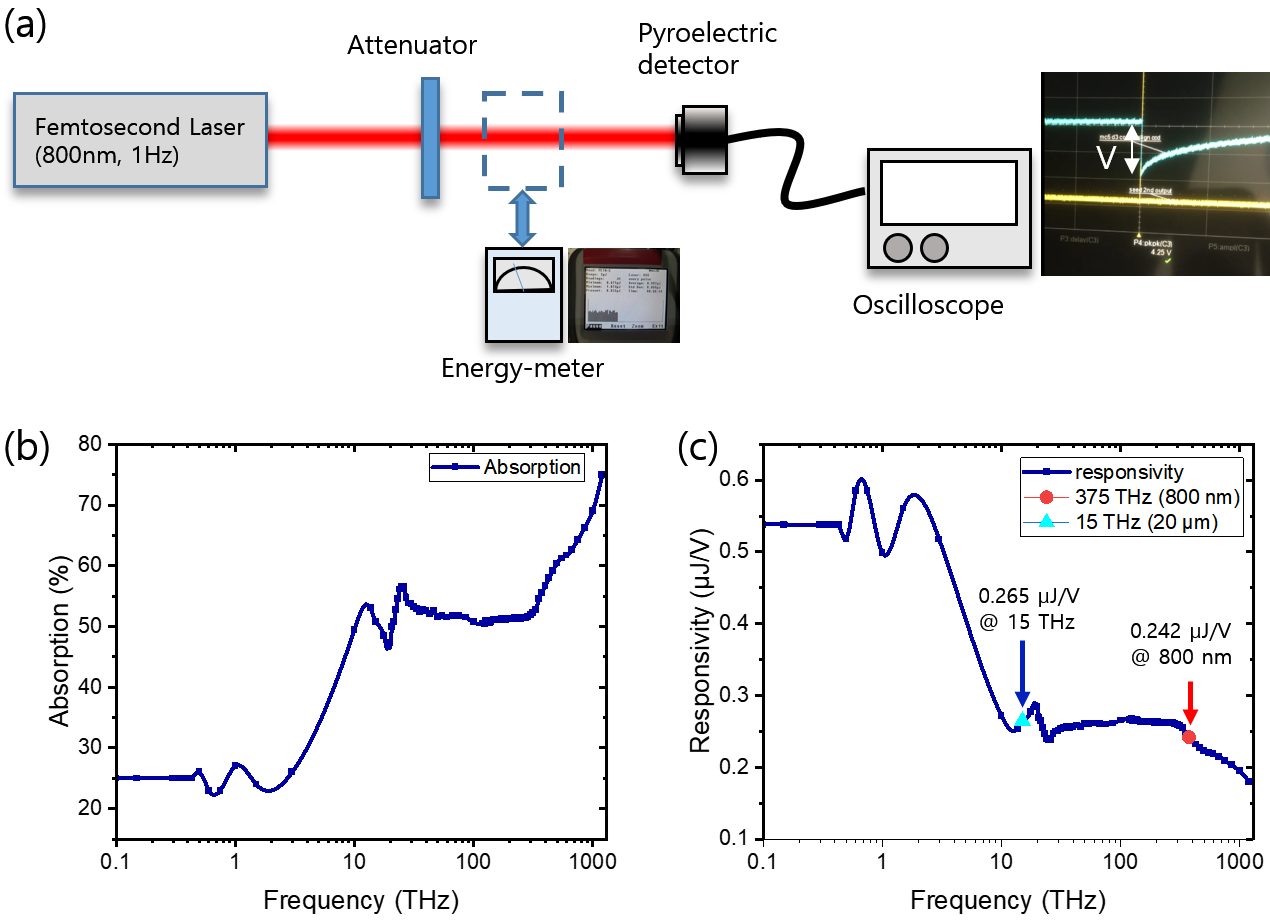


**Fig. S1.** **Responsivity characterization of the pyroelectric detector (Gentec, THz5D-MT-BNC).** **a** Experimental setup using a Ti:sapphire femtosecond laser with its repetition rate reduced at 1 Hz. The responsivity of the detector was measured to be 0.242 μJ V^-1^ at 800 nm. **b** Absorption of the chromium layer coated onto the detector’s lithium tantalate sensor, provided by the vendor (0.25 to 15 μm NIST Traceable; 15 to 440 μm Spectrometer Data; 440 to 3000 μm Guestimate). **c** Spectral responsivity curve of the detector calibrated using its responsivity at 800 nm. The detector’s responsivity at 20 μm (15 THz) is characterized to be 0.265 μJ V^-1^.


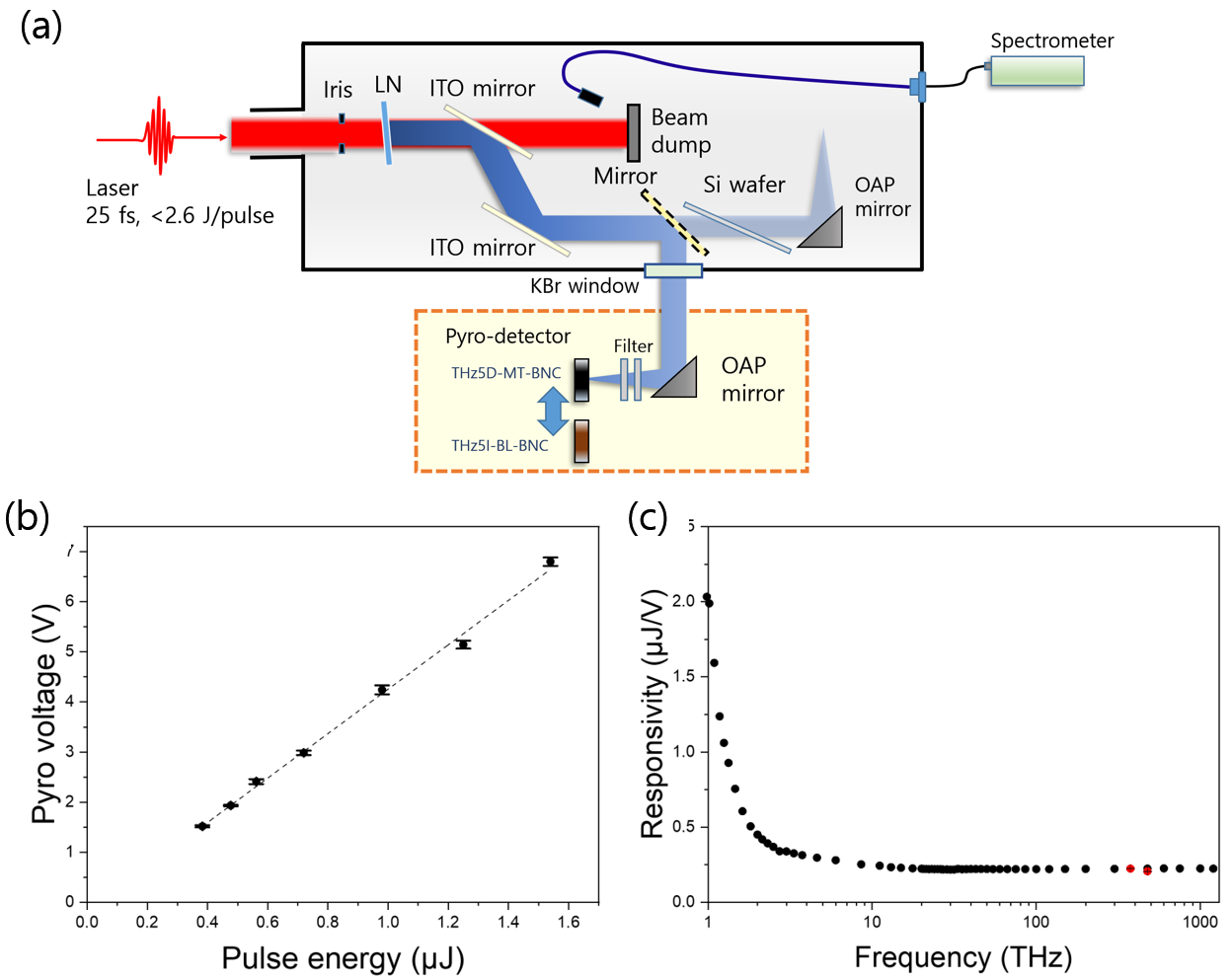


**Fig. S2.** **THz energy measurements with two types of pyroelectric detectors.** **a** Two lithium tantalate detectors, coated with chromium (Gentec, THz5D-MT-BNC) and organic black (Gentec, THz5I-BL-BNC), are used for cross checking in THz energy measurements. **b**, **c** THz energy and spectral calibration of the organic black detector (THz5I-BL-BNC). From the slope in (**b**), the responsivity is estimated to be 0.220 ± 0.008 μJ V^-1^ at 800 nm. In (**c**), the responsivity at 15 THz is expected to be 0.225 μJ V^-1^ from calibration data provided by the vendor. The organic black (OL) and chromium (MT) detectors provided 3.23 V and 2.95 V at laser energy of 2.57 J and 2.66 J, respectively. From this, the responsivity of the chromium one is extracted to be 0.255 μJ V^-1^ at 15 THz. This value is chosen for the responsivity of the detector (Gentec, THz5D-MT-BNC), instead of 0.265 μJ V^-1^ obtained in Fig. S1, to be more conservative in estimating THz energy.


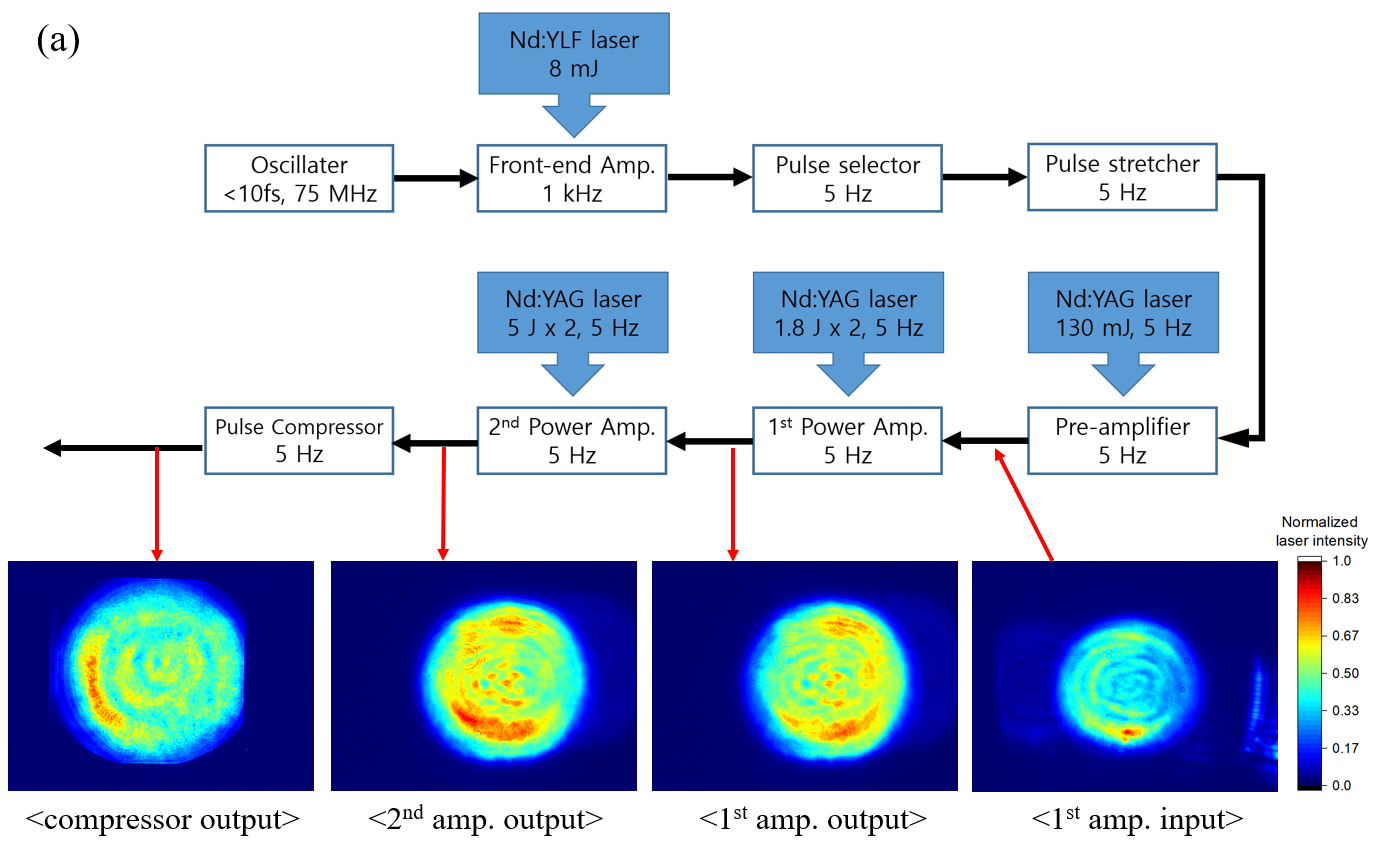


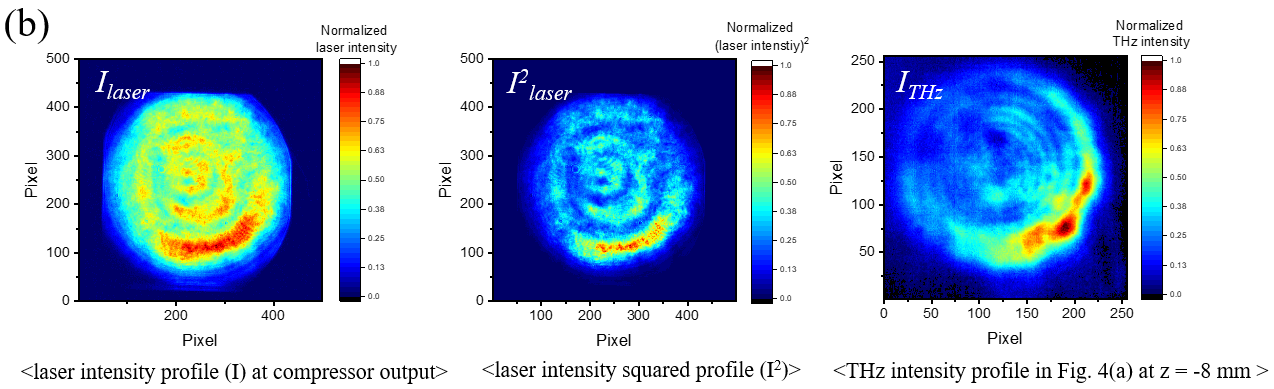


**Fig. S3. Laser and THz beam profiles. a** A schematic diagram of the 150-TW Ti:sapphire laser used in the experiment and laser beam profiles after each amplification/compression process. The laser consists of a 1-kHz multi-pass amplifier, a pulse stretcher, a pre-amplifier, two-stage amplifiers, and a pulse compressor. The final laser beam profile, taken immediately after the compressor, is considered to be similar to the real laser intensity distribution on the LN wafer. A nonuniform distribution is observed and expected to originate from the pump laser (Nd:YAG) whose beams are apertured by the crystal rods (12.7 mm in diameter). This results in the diffraction rings in the measured beam profiles. **b** Measured laser intensity (*I_laser_*, left), calculated laser intensity squared (*I^2^_laser_*, middle), and measured THz intensity (*I_THz_*, right) profiles. *I_THz_* is expected to be proportional to *I^2^_laser_* as optical rectification relies on the second order nonlinearity (*E_THz_* ∝ *E*^2^*_laser_*). The locally bright edge appearing in *I_THz_* also is observed in *I^2^_laser_*. Any difference between them can be explained by the fact that the laser intensity profile *I_laser_* was measured near the pulse compressor located ~11 m away from the LN emitter, and the beam profile was rotated due to the orientation of the camera.


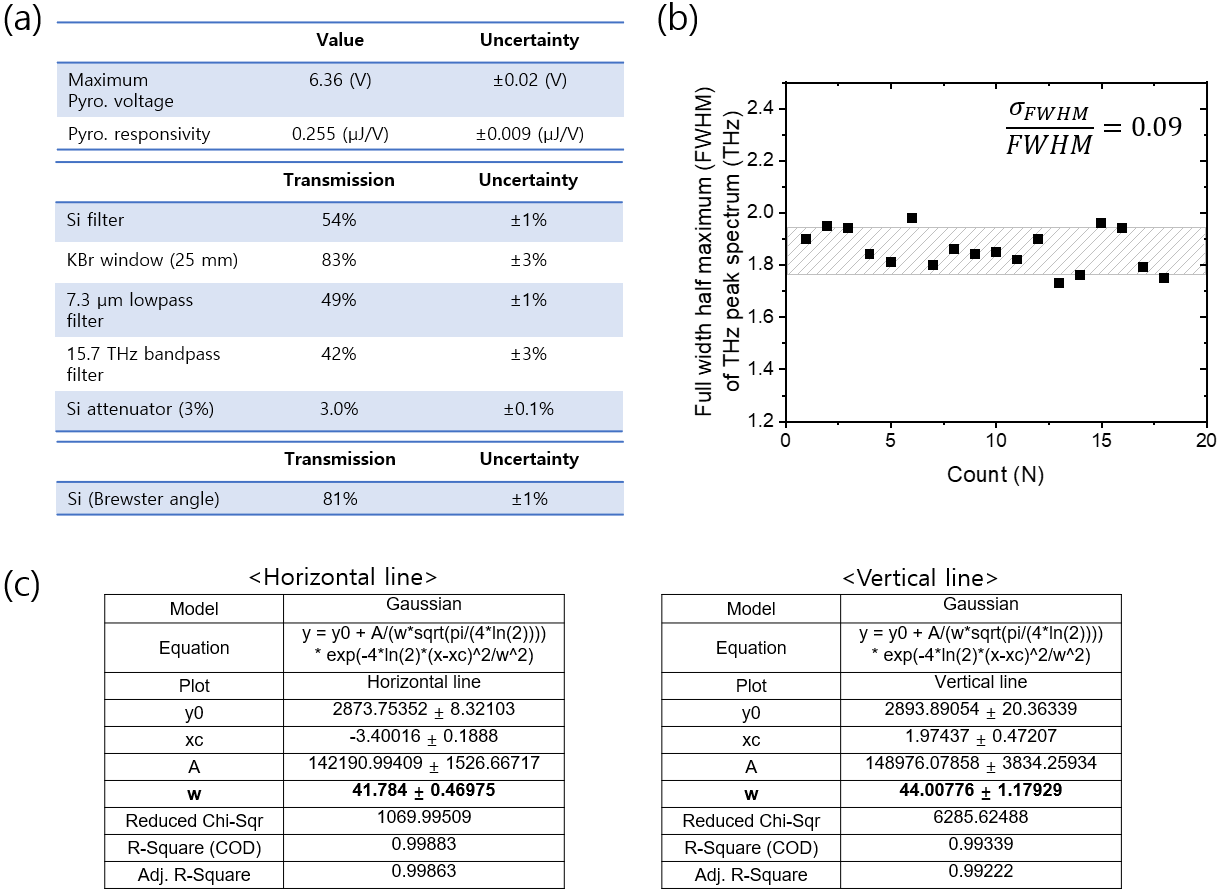


**Fig. S4. Uncertainties of the peak THz intensity and field strength values**: **a** The table lists all uncertainties in the measurements of the maximum pyroelectric signal, the pyroelectric responsivity, the transmission of all filters used for THz energy measurements (removed for maximal THz generation), and the transmission of the Brewster-angled Si (inserted for blocking laser leakage). This provides a peak THz energy of $\varepsilon$ = 0.47 ± 0.05 mJ at the focus. **b** Various THz spectral bandwidths extracted from multiple lines in the THz autocorrelation image in Fig. 5(a). This provides a Gaussian transform-limited pulse duration of *τ* = 0.23 ± 0.02 ps in FWHM. **c** Uncertainties of the parameters used for Gaussian fittings of the THz focal beam profiles (horizontal and vertical) in Origin. This provides *w_h_* = 41.8 ± 0.5 μm and *w_v_* = 44 ± 1 μm with a geometric average of *w*_FWHM_ = 43 ± 1 μm. The corresponding beam radius at 1/*e*^2^ is *w* = 37 ± 1 μm. Finally, the fractional uncertainties of the peak THz intensity (*I_T_*) and field strength (*E_T_*) can be estimated as

$$\frac{\sigma_{I_{T}}}{I_{T}}=\sqrt{\left( \frac{\sigma_{\varepsilon}}{\varepsilon} \right)^{2}+\left( 2\frac{\sigma_{w}}{w} \right)^{2}+\left( \frac{\sigma_{\tau}}{\tau} \right)^{2}}\sim0.15,$$

$$\frac{\sigma_{E_{T}}}{E_{T}}=\frac{1}{2}\frac{\sigma_{I_{T}}}{I_{T}}\sim0.08.$$

This gives a peak intensity of (8.9 ± 1.3)×10^13^ W cm^-2^ and a peak electric field strength of 260 ± 20 MV cm^-1^.


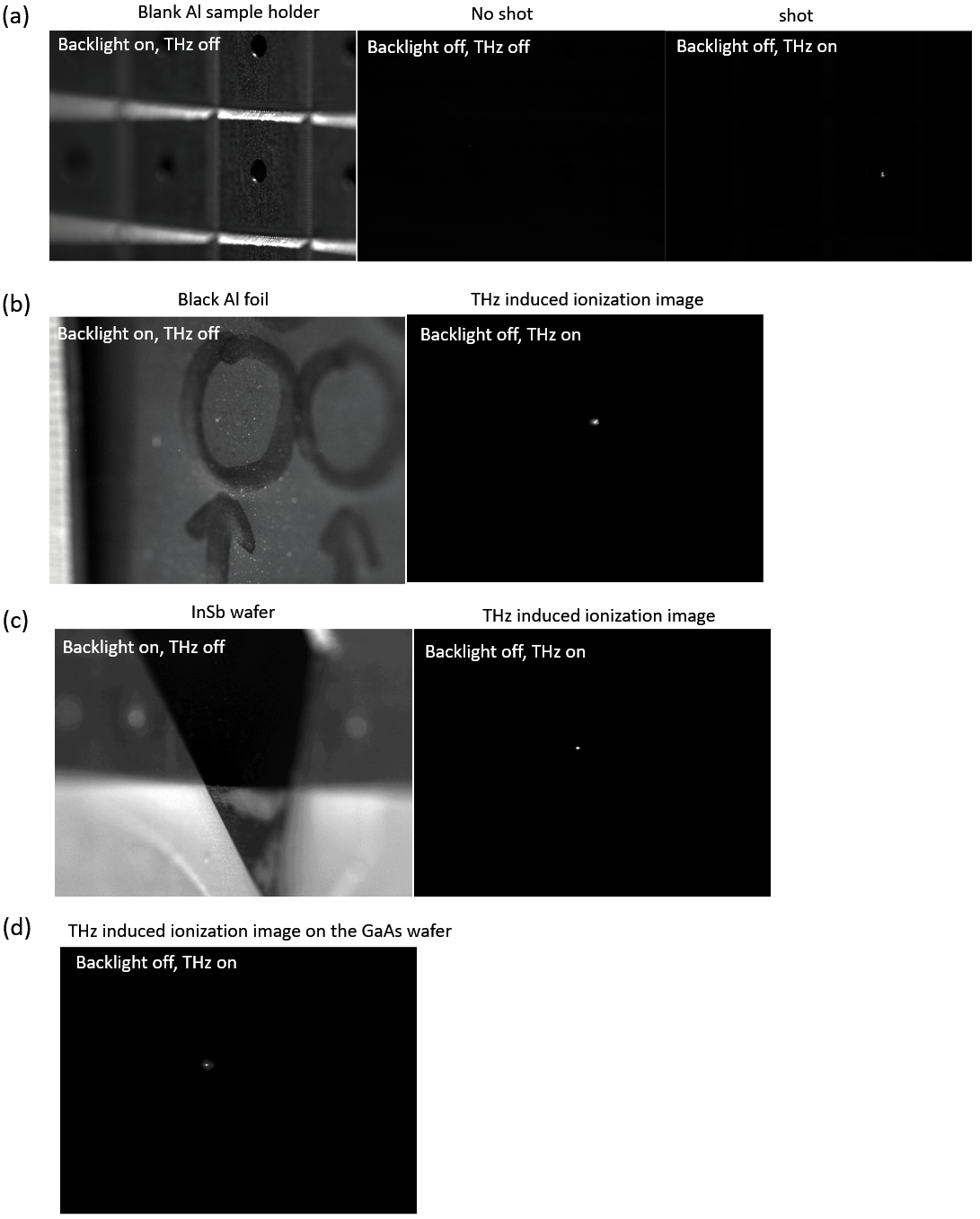


**Fig. S5.** **Raw CCD images of target samples and plasma fluorescence induced by THz radiation.** The samples include (**a**) a blank aluminum (Al) sample holder, (**b**) a black Al foil, (**c**) an InSb wafer, and (**d**) a GaAs wafer.
